# Dissemination of an evidence-based motivational interviewing brief intervention for substance use disorders to HIV service organizations across the United States: protocol for a national-level cluster-randomized adaptive parallel-groups superiority experiment

**DOI:** 10.1186/s13722-025-00612-8

**Published:** 2025-10-23

**Authors:** Hannah K. Knudsen, Heather J. Gotham, Elizabeth Solinger, Elizabeth Swan, Jen Brinker, Sheila V. Patel, Stephen J. Tueller, Michael Bradshaw, Jackie Mungo, Sarah Philbrick, Tom Donohoe, Thomas E. Freese, Beth A. Rutkowski, Mathew R. Roosa, Kathryn J. Speck, Bryan R. Garner

**Affiliations:** 1 University of Kentucky, 845 Angliana Avenue, Room 204, Lexington, KY 40508 USA; 2 Stanford University, 1070 Arastradero Rd, Palo Alto, CA 94304 USA; 3 The Ohio State University, 700 Ackerman, Columbus, OH 43221 USA; 4 RTI International, P. O. Box 12194, Research Triangle Park, NC 27709-2194 USA; 5 University of California, Los Angeles, 10911 Weyburn Avenue, Suite 200, Los Angeles, CA 90024 USA; 6 Roosa Consulting, Beacon, NY USA; 7 University of Nebraska Public Policy Center, 215 Centennial Mall South, Suite 401, Lincoln, NE 68588 USA

**Keywords:** Dissemination, Exploration, Adoption, Facilitation, HIV, Substance use disorders, Motivational interviewing, Brief intervention

## Abstract

**Background:**

Given the high prevalence of substance use disorders (SUDs) among people with HIV, integration of evidence-based SUD services within HIV service settings is needed. Federally-funded training and technical assistance centers, such as the Health Resources and Services Administration-funded AIDS Education and Training Center (AETC) network, are part of the support system that deliver strategies to promote the uptake of evidence-based interventions. Previously, our program of research supported (1) the effectiveness of facilitation for improving both the implementation and effectiveness of a motivational interviewing-based brief intervention for SUDs in HIV service organizations (HSOs), (2) motivational interviewing as having the best setting-intervention fit for HSOs, and (3) distributing educational materials as having the best setting-strategy fit for AETCs. Because exploration and adoption/preparation are phases that must occur before implementation and sustainment, we seek to test the effectiveness of facilitation to augment an initial distributing educational materials dissemination strategy.

**Methods:**

Using a multi-cohort cluster-randomized adaptive parallel-groups superiority experiment design, our aim is to test the effectiveness of augmenting a standard distributing educational materials dissemination strategy with a brief exploration facilitation dissemination strategy to support implementation of a motivational interviewing-based brief intervention. For each rollout, HSOs not responding to the distributing educational materials strategy will be randomized to either (a) receive no further intervention or (b) receive the exploration facilitation strategy. Assessed 4-months post-dissemination, the primary dissemination outcome is organizational-level adoption/preparation of the intervention (i.e., 1 + HSO staff downloads the manual and/or enrolls in the online asynchronous training). The secondary dissemination outcome is organization-level exploration of the intervention (i.e., 1 + HSO staff opens the dissemination email).

**Discussion:**

Before evidence-based interventions are implemented in practice to improve health outcomes, they must first be successfully disseminated and then explored and adopted by service organizations and providers. Because training and technical assistance centers have limited resources for dissemination efforts, effective adaptive dissemination strategies that utilize limited resources are needed. If we find the exploration facilitation strategy to be an effective adjunct to the distributing educational materials strategy, we hope that this adaptive dissemination strategy can be widely adopted and implemented by training and technical assistance centers.

**Trial registration:**

Open Science Framework: https://osf.io/urgzd. Registered 1/30/2025.

**Protocol version:**

1/30/2025 Version 1.

**Supplementary Information:**

The online version contains supplementary material available at 10.1186/s13722-025-00612-8.

## Background

People with HIV are at elevated risk of developing substance use disorders (SUD), with some estimates indicating nearly 50% of people with HIV have a SUD [1]. This prevalence rate is six times greater than the general United States adult population [2]. HIV service organizations (HSOs) are diverse in the services that they provide for people with HIV, with some largely providing clinical care and others largely providing case management focused on social determinants of health. HSO staff have reported high rates of specific SUDs among their clients, ranging from 28% for cocaine use disorder to 42% for alcohol and cannabis use disorders [3]. These rates are particularly concerning because SUDs negatively impact the HIV care continuum, including retention in HIV care, medication adherence, and viral load suppression [4–6].

Given these high rates of SUD, evidence-based interventions (EBIs) for SUD need to be integrated in settings that serve people with HIV [7–9]. However, scaling up EBIs requires attention to factors at the organizational and systems level. Organizational change theories and implementation science frameworks point to the importance of the fit between an EBI and the setting in which it is to be implemented [10–14]. However, setting-intervention fit has often been overlooked [15], which may impede dissemination and implementation efforts. Recently, setting-intervention fit for SUD service integration was studied through a stakeholder-engaged real-time Delphi of 202 HSOs. Of nine SUD interventions that included medications and behavioral therapies, motivational interviewing was the most promising EBI in terms of being fundable, implementable, retainable, sustainable, scalable, and timely, indicating setting-intervention fit [16]. Motivational interviewing is a patient-centered counseling approach that utilizes a facilitative style to help patients resolve ambivalence about behavior change [17]; compared to no treatment, motivational interviewing reduces substance use [18].

Of note, motivational interviewing has been adapted for the HSO setting. In a dual-randomized type 2 hybrid trial, Garner et al. [19] supported the effectiveness of a motivational interviewing-based brief intervention (MIBI) for clients with comorbid HIV and SUD. This trial also compared two implementation strategies, which are methods and techniques that help organizations to adopt and implement EBIs [20]. All HSOs received training, feedback, and consultation. Half of the HSOs also received facilitation, which is an implementation strategy that uses a process of interactive problem solving; it often involves an external facilitator who works with agency staff to identify supports for implementation efforts and strategies to overcome barriers [21]. Adding facilitation to a staff-focused training, feedback, and consultation strategy yielded greater implementation effectiveness, defined as quality and consistency, than training, feedback, and consultation [19].

Given evidence of the MIBI’s effectiveness and its setting-intervention fit, a key question is how to effectively disseminate this EBI to HSOs [22–25]. Dissemination, “the active approach of spreading evidence-based interventions to the target audience via determined channels using planned strategies” [26] (p. 339), is critically important to integrating EBIs and improving health outcomes [24, 27]. Dissemination strategies are most relevant during the early phases of moving an EBI into practice. The EPIS (exploration, adoption/preparation, implementation, sustainment) framework characterizes this process [28]. In the exploration phase, an organization realizes that their clients have an unmet need and considers EBIs to meet that need. Over time, an organization may move into the adoption/preparation phase, which entails identifying implementation strategies (e.g., training) to support EBI delivery [23]. Dissemination strategies that focus on raising awareness of an EBI and availability of training may help organizations to move through these early phases.

**Fig. 1:**
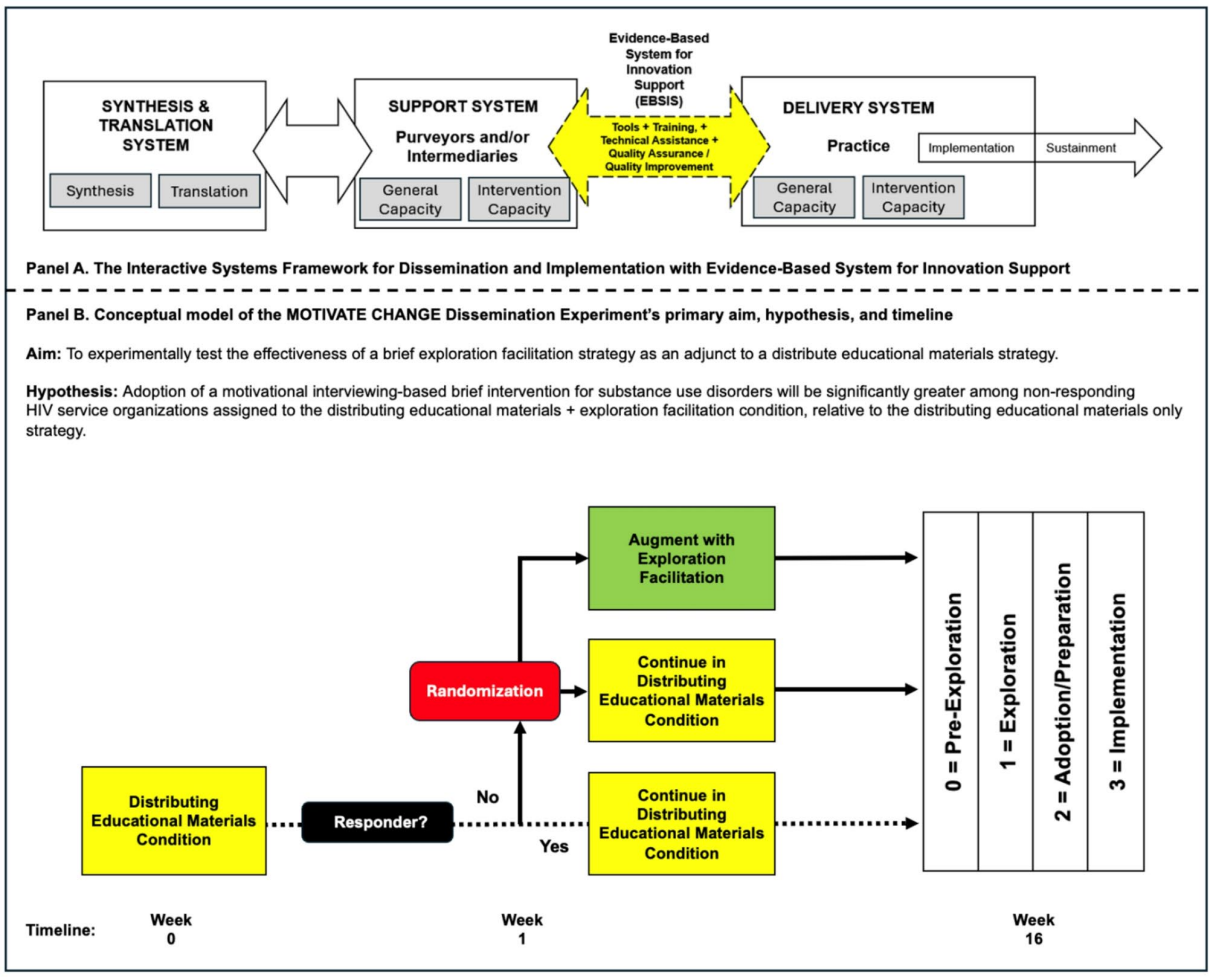
Guiding framework (panel A) and conceptual model of aim, hypothesis, and timeline (panel B)

A critical issue for scaling up EBIs is identifying effective dissemination strategies that can be utilized by existing entities that support dissemination and implementation efforts. As seen in Fig. 1, according to the Interactive Systems Framework for Dissemination and Implementation [29–31], intermediaries and purveyors of EBIs can play important roles in dissemination and implementation efforts. In the United States, there are federally-funded training and technical assistance centers, which provide training on EBIs as well as other implementation strategies [31]. It is important to design dissemination strategies that can accelerate EBI exploration and adoption/preparation while being feasible for training and technical assistance centers to provide.

For HSOs in the U.S. and its territories, the Health Resources and Services Administration (HRSA)-funded AIDS Education and Training Centers (AETC) are a support system that provide training and technical assistance. A recent real-time Delphi survey of 64 AETC representatives sought to identify the most promising dissemination and implementation strategies [21] using a setting-strategy fit index [32]. For each of ten strategies, representatives rated importance, feasibility, readiness, scalability, pressure to offer, and need. The strategies with the highest setting-strategy fit were distributing educational materials and providing access to asynchronous training.

This article describes the protocol for MOTIVATE CHANGE (Motivational Outreach and Training Interventions for Vulnerable Affected Targeted Empowerment, and Counseling for HIV-positive clients with Addiction Needs for Guidance and Education), a national-level cluster-randomized adaptive parallel-group (1:1 ratio) superiority experiment. The SPIRIT guidelines have been used to structure this protocol [33]. As shown in Fig. 1, the primary aim is to test the effectiveness of augmenting an initial distributing educational materials strategy (i.e., an email with key messages about the MIBI and an invitation to a website with access to a MIBI manual and other training resources upon enrollment) with a brief exploration facilitation strategy. The exploration facilitation strategy utilizes a trained facilitator who leads an agency representative through a discussion of supports and barriers to implementing the MIBI while addressing ambivalence about implementation using a supportive interpersonal style. For HSOs not responding to the initial strategy, the HSO (and its staff) will be randomized to receive either (a) an exploration facilitation strategy or (b) no further intervention. Distributing educational materials was selected as the control strategy because it had the greatest setting-strategy fit in our survey of AETCs [32]. Exploration facilitation was selected as the experimental dissemination strategy based on implementation research supporting it as an effective strategy when used during the preparation, implementation, and sustainment phases [19]. The primary hypothesis is that for HSOs that do not respond to the initial distributing educational materials dissemination strategy, HSO-level MIBI adoption (i.e., HSOs with 1 + staff downloading the MIBI manual and/or enrolling in the online training) will be significantly greater among HSOs assigned to the distributing educational materials plus exploration facilitation condition than HSOs in the distributing educational materials-only condition. As such, this study seeks to help address the need for rigorous research on dissemination strategies, a need most recently underscored by Turon and colleagues [25] as part of their scoping review on dissemination of public health research to prevent non-communicable diseases.

## Methods/design

### Study setting

The MOTIVATE CHANGE dissemination experiment will be conducted by recruiting community-based HSOs and their staff located in the contiguous United States and District of Columbia. HSOs vary in services provided, as some are primarily clinical (e.g., prescribing HIV medications, providing CD4 T-lymphocyte testing and HIV viral load testing), and others are primarily non-clinical (e.g., providing medical and non-medical case management to people with HIV to address social determinants of health).

### Eligibility criteria

A national directory of HSOs developed for two earlier studies was updated to identify potentially eligible HSOs. In 2018–2019, research staff created a comprehensive database of HSOs via publicly existing resources (e.g. HIV.gov, poz.com, thebody.com) and then called each HSO to confirm they were open and update contact information [3]. This process was repeated in 2020 [16] and in 2023 for the current study. HSOs must be open or perceived to be open at the time of the experiment launch and must provide HIV-related services such as case management, direct healthcare, behavioral health services (including to address substance use), or linkage to care. Determinations regarding eligibility are made based on organizational websites. HSOs without a functional website are considered “closed” and excluded. HSOs that had previously participated in the SAT2HIV or SAT2HIV-II studies, which focused on implementation strategies for the MIBI, are excluded (*n* = 43).

For eligible HSOs, organizational websites are searched to identify email addresses of staff members who interact with clients or individuals who make decisions regarding staff training, such as chief executive officers, office administrators, clinic managers, or education/training managers (e.g., leadership, staff, or staff directory sections). Individuals in these roles are added to the study database. To increase the validity and deliverability of the study-related emails, HSO Directory email addresses are evaluated using Bouncer, a paid online email validation service that checks activity and the ability to receive messages. Email addresses deemed deliverable, risky, or unknown are included in the experiment; undeliverable email addresses are excluded.

Provider-level eligibility criteria for MOTIVATE CHANGE are being: (1) a staff member of an agency in the HSO Directory located in the contiguous US states (residents of Alaska and Hawaii are not eligible due to limitations of gift card distribution) or District of Columbia; (2) at least 18 years of age; (3) fluent in English;, and (4) willing and able to provide consent to participate in the study. Any provider who meets inclusion criteria can participate, so there is no pre-set numbers of providers allowed per agency.

### Strategies

An overview of the two dissemination strategies being tested is provided in Table 1 using the Strategies Timeline, Activities, and Rationale (STARationale) Table format. STARationale was recently introduced by Garner et al. [34] to help standardize the specification and reporting of strategies in accordance with recommendations by Proctor et al. [35] and Perez-Jolles et al. [36]. Additional details regarding each strategy, which are delivered by the study team, are provided in the text below and in Table 1.

**Table 1:** The strategies timeline, activities, and rationale (STARationale) table for the MOTIVATE CHANGE project

	STRATEGY ACTIVITIES			
**STRATEGY** ***TIMELINE***	**Operationalization** (i.e., name and general description/definition)	**Function** (i.e., purpose and targeted implementation or client outcomes)	**Form** (i.e., a precise description of the activity, delivering actor, and dose)	**STRATEGY** ***RATIONALE***
*1st Strategy*	Distributing educational materials. A staff-focused strategy that seeks to give HSO staff access to MIBI-related educational materials (e.g., manual, online course)	To help HSO staff adopting the MIBI learn how to implement the MIBI with clients that are in need.	An initial email (see Supplemental File 1) and one reminder email, each sent by the research team email.	Powell et al. [28] recommended and Patel et al. [29] found to have the greatest setting-strategy fit.
*2nd Strategy*	Exploration Facilitation. A staff-focused strategy that uses principles of motivational interviewing to help increase the likelihood of MIBI adoption by one or more HSO staff.	To help increase the likelihood of exploration and adoption/preparation by HSO staff who did not respond to the distributing educational materials strategy.	Up to three telephone call attempts by a trained facilitator to engage, focus, evoke, and plan with an HSO staff regarding exploration and adoption/preparation of the MIBI.	Powell et al. [28] recommended and Garner et al. [17, 34–36, 43] supported facilitation as an effective implementation strategy.

Note: AETCs = AIDS Education and Training Centers; HSO = HIV Service Organization; MIBI = Motivational Interviewing-based Brief Intervention

#### Distributing educational materials

All HSOs begin in the distributing educational materials condition and receive the MOTIVATE CHANGE dissemination email (see Supplemental File 1), which has been designed to deliver three key messages: 1) SUD is common among people with HIV; 2) the MIBI, which takes 15–30 min to deliver, reduces substance use; and 3) the study is offering no-cost access to training resources on the MIBI. The dissemination email was developed using feedback from the study’s guiding coalition comprised of experts in HIV and SUD. Rolling batches of HSOs will be constructed for study management purposes. For each batch, HSO staff will be initially emailed on Tuesday. If a staff member from the HSO does not enroll by Friday, a dissemination email reminder will be sent. Email recipients have approximately 30 days to engage with the study’s landing page and enroll in the study by clicking the email’s “Learn More and Enroll” button before their access to the landing page expires. A one-week run-in period will be used to determine the percentage of HSOs for which the distributing educational materials emails is sufficient to move at least one HSO staff from pre-exploration to exploration, defined as at least one staff opening either of the dissemination emails. HSOs with at least one staff member enrolling in the study during the run-in period will not be randomized and will be categorized as a separate group (“immediate exploration”).

HSO staff who click the “Learn More and Enroll” button in the dissemination email will be taken to the study’s landing page to provide electronic informed consent, complete a brief baseline demographics survey, and gain access to Phase I resources for approximately thirty days from the time of the initial email invitation. Phase I resources include the MIBI Manual for self-review and no-cost access to the online course, “A Tour of Motivational Interviewing: An Interprofessional Road Map for Behavior Change” (Tour of MI). The MIBI Manual is a comprehensive guide that outlines the MIBI intervention, provides practical tools for conducting the intervention, and requires less than 90 min to review. Tour of MI is a self-paced, four-hour online course developed by the Mid-America Addiction Technology Transfer Center at the University of Missouri-Kansas City School of Nursing and Health Studies. This course offers foundational training in MI, emphasizing collaborative conversations to strengthen a client’s motivation for behavior change and the use of MI techniques within an atmosphere of acceptance and compassion. The course content is applicable across health professionals. Some professions are eligible for 4.0 continuing education credits for a fee (CE; Continuing Medical Education, Continuing Nursing Education, National Association of Social Workers, and NAADAC); a certificate of completion is available if CEs are not provided. If the participant provides documentation confirming prior completion of the Tour of MI, they will be fast-tracked to Phase II.

In Phase II, staff participants who complete the Tour of MI will receive no-cost access to two online learning platforms (SIMmersion and LYSSN) for 60 days. SIMmersion is an interactive simulation platform that facilitates the development of MI communication skills through practice with virtual role-players simulating SUD-specific scenarios. Participants are provided instant feedback, performance evaluations, and suggestions for improvement by an on-screen virtual coach to help them refine their MI skills. Participants can track their progress and identify areas for development. LYSSN, an AI-driven training platform, provides participants with on-demand practice and feedback on their MI sessions. LYSSN generates transcripts and expert analyses of training sessions, assessing the quality and effectiveness of the MI sessions. The platform offers real-time, actionable feedback based on over 55 fidelity measures. Participants will access SIMmersion and LYSSN through individual accounts managed by the study team.

#### Exploration facilitation

Following the initial one-week run-in period, approximately half of the non-responding HSOs will be randomized to receive the project’s exploration facilitation dissemination strategy. The rationale for testing exploration facilitation as a dissemination strategy is provided by research supporting the effectiveness of the implementation and sustainment facilitation (ISF) strategy [19, 37–40]. For HSOs and their staff randomized to the distributing educational materials + exploration facilitation strategy, an external facilitator on the study team who is trained in ISF Strategy and motivational interviewing (MI) will call the HSO within ~ 10 business days after randomization and attempt to reach a training manager or other appropriate representative. Facilitators will make up to three attempts to contact an appropriate staff member at the HSO and a voicemail may be left requesting a return call. HSO staff will also have the option to schedule a facilitation call if the time of initial contact is not convenient.

The exploration facilitation strategy is estimated to take up to 30 min. The call will begin with an introduction and verbal informed consent. General HSO questions will be asked, such as organizational ownership, number of staff, number of clients with HIV, and current use of SUD screening and SUD interventions. Then, an overview of the MIBI and the study will be provided. The trained facilitator will then lead the participant through an unscripted decisional balance exercise to assess supports and barriers to implementing the MIBI at their HSO. Through this MI-based exercise, facilitators will seek to help participants find the motivation to change the practices at their HSO by addressing ambivalent feelings and uncertainties. The facilitator will resend the original dissemination email to the participant so that prior to expiration they may review the landing page on their own time and proceed to study enrollment if they choose. Access to the training resources remains on the original schedule (i.e., access expires approximately thirty days from the date of the initial email invitation). The facilitator will also encourage the participant to forward the dissemination email to other HSO staff.

#### Control condition

For HSOs that do not respond to the initial distributing educational materials strategy and are randomized to the control condition, no additional strategies will occur. If staff in control HSOs or immediate exploration HSOs click on the dissemination email before their landing page access expires (i.e., approximately thirty days after first dissemination email is sent), they will have access to the Phase I training tools (described above) and can proceed to Phase II if they complete the Phase I requirements.

### Outcomes

**Fig. 2:**
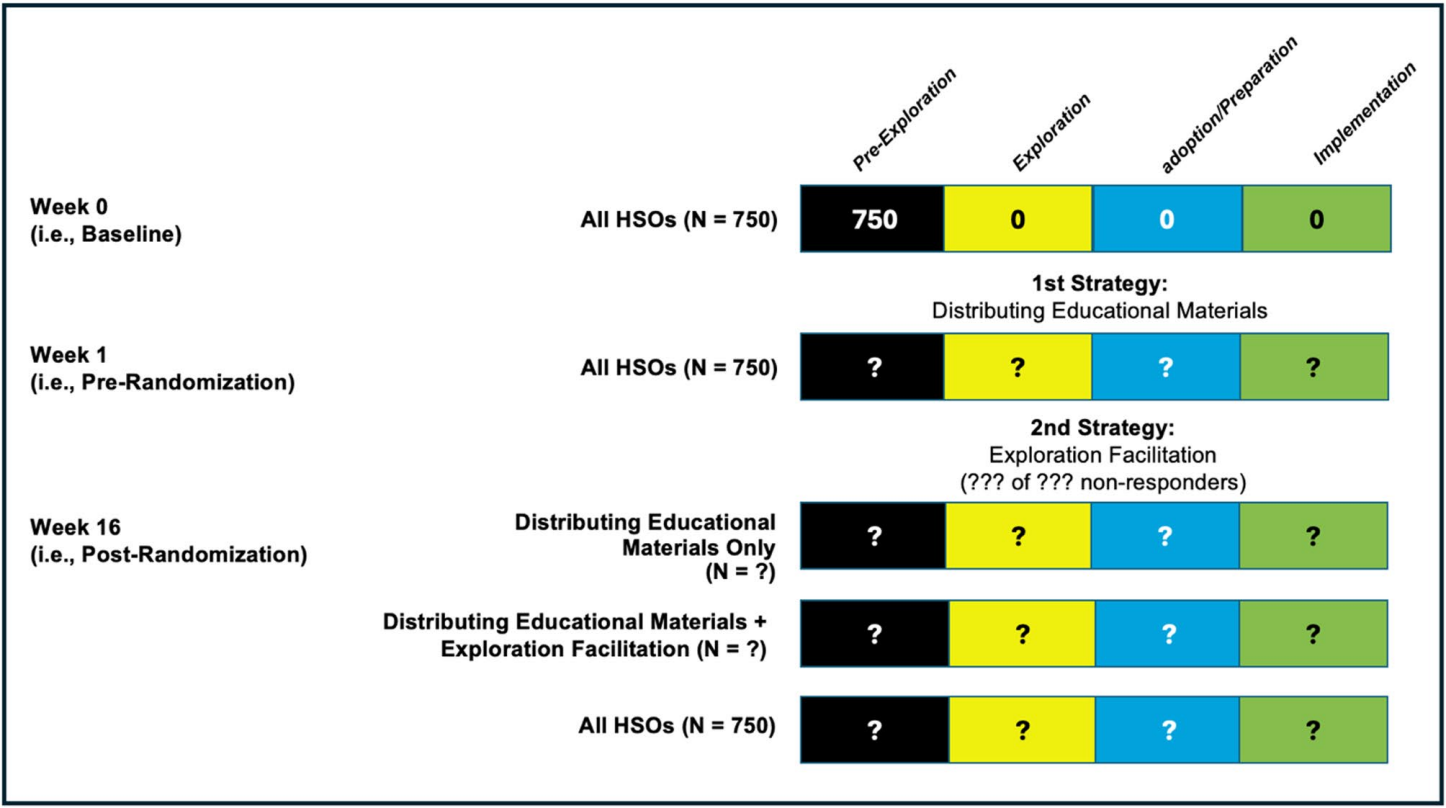
Repeated status assessment for motivational interviewing brief intervention

Guided by the extant literature, our primary dissemination outcome is *adoption*, which Baumann et al. [24] define as occurring when “the individual or organization engages in a number of activities that will lead to the research evidence being integrated into clinical practice and/or policy decisions” (p. 8). Per this definition and our cluster-randomized design, we will examine both *staff-level MIBI adoption* (i.e., number of HSO staff who download the MIBI manual and/or enroll in the online asynchronous MI training) and *organization-level MIBI adoption* (i.e., number of HSOs with staff-level MIBI adoption by 1 + staff). Secondary outcomes are: *staff-level MIBI exploration* (i.e., number of HSO staff who open either dissemination email), *organization-level MIBI exploration* (i.e., number of HSOs with staff-level MIBI exploration by 1 + staff), *staff-level MIBI implementation* (i.e., number of HSO staff who self-report any use of the MIBI with clients at the four-month follow-up survey), and *organization-level MIBI implementation* (i.e., number of HSOs where at least one staff person reports any use of the MIBI with clients at the four-month follow-up survey). Figure 2 illustrates MIBI status progression at the HSO-level. For this study, initial MIBI status will be Pre-Exploration for all HSOs and their staff. Transition to other statuses will be based on information from the project’s enrollment database and four-month follow-up survey using the outcome definitions above.

### Participant timeline, sample size, and recruitment

Figure 1 provides an overview of the participant timeline. Regarding sample sizes, we anticipate approximately 750 HSOs and 3,100 individual HSO staff will eligible and have the opportunity to enroll in the study. Regarding recruitment, HSO points of contact will be sent the initial dissemination email (see Supplemental File 1) via Salesforce in a staggered batch format of up to 180 HSOs in six separate batches. The dissemination email will link to a landing page with information about the study and MI. Participants may choose to click the “Learn More & Enroll” button and be directed to the REDCap (Research Electronic Data Capture) [41] study consent and demographics survey.

### Assignment of strategies

#### Allocation

Randomization will occur at the HSO-level among HSOs with no staff enrolled in the study at the end of the initial distributing educational materials run-in period. HSOs will be allocated in a 1:1 ratio to the distributing educational materials + exploration facilitation condition or to remain in distributing educational materials only condition. A randomization table will be created by the project’s statistician (ST) prior to the start of the study, and as HSOs enroll, they will be allocated to study condition using the next row in the randomization table.

#### Blinding (Masking)

HSO staff will not be blinded to study condition. Individuals assigning the scores on the outcomes will be blinded to study condition.

### Data collection and management

#### Staff surveys

Staff-level data collection occurs at two timepoints: baseline and four-month follow-up. After providing online consent, HSO staff will be asked to complete a brief baseline survey via REDCap that includes questions about demographics (age, race, ethnicity, sex assigned at birth, gender, educational attainment, certification/licensure), years of experience working at HSOs, tenure at current HSO, and experience with MI. The three experience questions ask staff to self-report their (a) familiarity (“not at all familiar,” “somewhat familiar,” “moderately familiar,” or “extremely familiar”), (b) experience (“novice,” “moderate,” or “experienced”), and (c) training (checking all that apply from “professional in person conference or training,” “online course or training,” “work mandated training,” “self-taught from literature,” and “another setting” with a text box to describe) related to using MI with clients who have risky substance use behaviors.

Approximately 4 months after the original dissemination email is sent, HSO staff enrolled in the study will receive an invitation to complete a follow-up survey via email. The invitation will direct participants to a REDCap survey that focuses on MIBI implementation and training resource use. The item measuring MIBI implementation asks, “In the last three months, have you completed a MIBI session with a client who displays risky substance use?” with response options of “yes,” “no,” and “unsure.” Responses of “yes” will be coded as MIBI implementation at the staff- and HSO-level. The survey then asks about the use of the four training resources that were made available (MIBI Manual, Tour of MI, SIMmersion, LYSSN). For each resource used, the survey includes five items measuring (a) relevance to the individual’s work, (b) learning how to apply MI to substance use, (c) ease of understanding the content, (d) feelings of preparation to use MI for substance use, and (e) learning foundational principles of MI. These items use a four-point Likert scale ranging from “strongly agree” to “strongly disagree.” For the MIBI Manual, additional questions ask about use of specific worksheets/tools with clients (“yes,” “no,” or “unsure”). For each resource not used, the survey assesses potential barriers: (a) pre-existing confidence in using MI for substance use, (b) competing work demands, and (c) lack of clients with risky substance use. For non-use of the MIBI Manual, additional barriers include manual length and lack of interest. Individuals not using Tour of MI will also be asked about the training being too long and previous completion of a similar training. Additional barriers specific to the two learning platforms will be included, such as not liking virtual simulations as a training method, perceived complexity of the websites, and not wanting feedback on MI skills. All items measuring potential barriers use a four-point Likert scale from “strongly agree” to “strongly disagree.” Participants who complete the follow-up survey will receive a $20 electronic gift card distributed by Virtual Incentives as compensation for their time.

#### Website usage data

The study team will obtain usage data for Tour of MI, SIMmersion, and LYSSN. For Tour of MI, data on completion of each module, full course completion, and quiz scores will be linked to the individual HSO staff member’s study record. Study team members will track the creation of individual accounts for SIMmersion and LYSSN. SIMmersion provides total minutes, level of proficiency, and scores for each simulation session; individual-level averages of proficiency and scores across sessions will be calculated. LYSSN provides transcripts of users’ interactions with each virtual simulation as well as counts of listening statements, motivating questions, affirmations, closed questions, giving information, advising, facilitating, and confrontations. Individual-level averages of MI-consistent and MI-inconsistent statements across sessions will be calculated along with changes in these statements from first to last session.

#### Data management

Data will be extracted from the REDCap system to develop analytic datasets. Logical checks and descriptive analyses will be used to assess errors, inconsistencies, and outliers. As part of the data management process, all constructed variables will be produced (e.g., scale scores, total scores). Distributional properties of outcome variables will be assessed to inform decisions about statistical models. Documentation will be prepared to accompany archived copies of the data. Finally, patterns of missing data will be evaluated. If there is evidence that data are not missing completely at random [42], methods for addressing missing data will be used as described in the section on statistical methods.

### Statistical methods

Statistical analysis of study outcomes will be conducted using an intention-to-treat approach, meaning all HSOs will be analyzed in the group they were randomized to regardless of whether they receive the exploration facilitation strategy. The primary effect to be estimated for each outcome is its difference between the three study groups (distributing educational materials only, distributing educational materials plus exploration facilitation, and immediate exploration). Prior to estimating effects between these groups, balance on demographic characteristics, HSO characteristics, and other background covariates will be assessed by estimating propensity scores between the immediate exploration group and the combination of the two randomized groups (which may have substantial differences), and between the two randomized groups (which should not have differences if randomization worked as expected). If substantial imbalance is observed, doubly robust effects will be estimated by including both propensity score weights and including aforementioned covariates in the models described next.

Since they were not randomized, the comparability between the distributing educational materials only condition participants and the immediate exploration participants will be evaluated prior to propensity score estimation. Comparison of the distributing educational materials plus exploration facilitation group and distributing educational materials-only control group are causal due to the randomization, while comparison of either of these groups to the immediate exploration group is subject to selection bias. However, this selection bias is in itself interesting, and the analysis results will be used to understand what predicts immediate engagement. Each outcome’s distribution will be assessed to ensure adequate distributional assumptions are made in statistical modeling. If outcomes exhibit strong floor and/or ceiling effects, they can be rescaled to a proportion scale and modeled with zero- and/or one-inflated beta regression [19, 43]. If the missing data analyses uncover issues that should be addressed, we will use either full information maximum likelihood or multiple imputation [42]. In addition, summary statistics will be calculated for individual-level follow-up survey data and usage data for Tour of MI, LYSSN, and SIMmersion.

### Ethics and dissemination

#### Research ethics approval

The MOTIVATE CHANGE dissemination experiment was reviewed and approved by The Ohio State University IRB (Protocol 2024B0200), under Federalwide Assurance No. FWA00006378 from the U.S. Department of Health and Human Services’ Office for Human Research Protections. Any protocol modification that affects study conduct, potential risks to participants, or safety of participants requires a protocol amendment, which will be submitted to The Ohio State University’s IRB for approval. No amendments will be implemented until IRB approval has been received.

#### Monitoring

The Principal Investigator (BG) assumes ultimate responsibility for study data and safety monitoring. The risks to participants in this study are minimal, however, there is always a potential risk of a data breach on any study platform or database. Thus, all efforts will be made to ensure the confidentiality and security of participant data. The Ohio State University’s IRB conducts annual and random audits to assess adherence to federal human subjects protection regulations and to ensure that the rights and welfare of human subjects are protected.

#### Consent

Informed consent will be obtained from all study participants. The dissemination emails will direct potential participants to the study’s landing page, which will provide brief descriptions of the study. The page will outline access to Phase I resources, including the MIBI Manual and the Tour of MI, and to Phase II platforms, SIMmersion and LYSSN. If the individual clicks the “learn more & enroll” button, they will be directed to the consent form. The consent form will include information on participants’ rights, as well as details on the data to be collected, such as a brief baseline survey, analytics from the Tour of MI, LYSSN, and SIMmersion, and a four-month follow-up staff survey. Individuals who chose the “I consent” option will be enrolled in the study. For HSOs randomized to distributing educational materials plus exploration facilitation due to non-response to the dissemination email, a verbal consent script will be used before starting the exploration facilitation call; individuals who visit the study’s landing page after the facilitation call will receive the same consent process as those who accessed the landing page via the dissemination email. Individuals cannot participate in any aspect of the project without first providing informed consent.

#### Confidentiality

Data provided as part of the study are confidential and will not be shared with anyone outside the study team. Efforts to protect participant confidentiality include: (a) assigning a unique participant number that is only accessible to the Principal Investigator and research coordinators, (b) securely storing all study documents (i.e., on a password-protected server located in a secure building), and (c) not including any identifying information in presentations or publications of study findings. De-identified data will be retained electronically by The Ohio State University until the final publication of associated study papers. Associated learning platforms are permitted to retain de-identified data indefinitely to be used to improve the operation and functionality of their products.

#### Declarations of interests

The authors declare that they have no competing interests.

#### Access to data

During the active data collection and analysis phase, access to data is restricted to the Principal Investigator, research coordinators, lead statistician, and statistical programmers. Following completion of the study and publication of primary findings, a de-identified dataset will be constructed for public access. This public access dataset will be archived in the National Addiction & HIV Data Archive Program.

#### Dissemination policy

Study findings will be disseminated regardless of whether or not the hypotheses are supported. The dissemination of findings will include presentations at professional conferences and publications in peer-reviewed scientific journals. To ensure the widest access possible, we will prioritize scientific journals with open access, which will also facilitate compliance with the NIH Public Access Policy. Additionally, the Principal Investigator will disseminate copies of presentations and publications to NIDA, HRSA, and AETCs.

## Discussion

Before evidence-based interventions are implemented in practice to improve health outcomes, they must first be successfully disseminated to and explored and adopted by service organizations and providers. This protocol paper describes the MOTIVATE CHANGE dissemination experiment, a national-level cluster-randomized adaptive parallel groups superiority experiment to test an exploration facilitation dissemination strategy as an adjunct to increase adoption/preparation (i.e., primary outcome) and exploration (i.e., secondary outcome) of an evidence-based MIBI for SUD beyond that achieved by an initial distributing educational materials strategy. In comparison to implementation research, there is a dearth of dissemination research, especially dissemination research experimentally testing dissemination strategies [25]. As such, MOTIVATE CHANGE helps address a significant gap in the current dissemination and implementation literature.

The MOTIVATE CHANGE dissemination experiment builds upon the hybrid effectiveness-implementation research experiments that our study team has completed, which support the MIBI as an effective intervention for helping address comorbid HIV and SUD [19, 44] and facilitation as a cost-effective implementation strategy for implementing the MIBI [38]. It also builds on our research to better understand the prevalence and negative impacts of comorbid HIV and SUD [3], the SUD-focused EBIs that are most promising for integration within HSO settings [16], and the strategies that are most promising for purveyors/intermediaries to use for supporting dissemination and implementation of SUD-focused EBIs in HSO settings [32]. Building on this program of research, the MOTIVATE CHANGE dissemination experiment will help us understand the current market viability/demand for the MIBI within HSO settings, which Proctor et al. [45] noted as a neglected concept in dissemination and implementation science, and the extent to which a brief exploration facilitation strategy can increase MIBI exploration and adoption/preparation beyond an initial distributing educational materials strategy.

Because training and technical assistance centers have limited resources for dissemination efforts, effective dissemination strategies that help them utilize their limited resources most efficiently are needed. The MOTIVATE CHANGE experiment’s adaptive design may help in this regard. By quantifying HSOs’ progress through exploration and adoption/preparation phases for the distributing educational materials strategy, results may help training and technical assistance centers to estimate how much change occurs in the very short-term (i.e., one week) versus a longer period (i.e., four months). Distributing educational materials via email invitations to web-based resources is a low-intensity strategy that should have minimal costs. Exploration facilitation has greater costs because of the time required for training facilitators and then delivering the strategy. However, some efficiency may be gained by targeting exploration facilitation to those HSOs for which distributing educational materials was insufficient, rather than offering exploration facilitation to all HSOs. Using an adaptive approach may save resources, which is important for training and technical assistance centers that often serve a large geographic region but have limited financial resources [46].

We estimate MOTIVATE CHANGE will disseminate the MIBI to approximately 750 HSOs. According to Rogers [14], “a six-month communication intervention might increase knowledge of an innovation on the part of 35% of the audience while influencing only 2 or 3% of the individuals to adopt the new idea” (p. 198). We may then expect our initial distributing educational materials strategy to lead to exploration by 263 HSOs (35% of 750) and adoption/preparation by 15 to 23 HSOs (2–3% of 750). Ideally, MOTIVATE CHANGE will achieve an overall MIBI adoption/preparation of 20% or more of the approximately 750 HSOs (*n* = 150), which Rogers [14] termed the “tipping point” and when reached may cause the rate of subsequent adoption/preparation to rapidly accelerate throughout the target audience. More realistically, however, MOTIVATE CHANGE will achieve MIBI adoption/preparation among only innovators and early adopters, which together represent the first 16% (2.5% innovators and 13.5% early adopters).

### Strengths and limitations

The MOTIVATE CHANGE dissemination experiment has strengths and limitations. Strengths include: (1) its national-level scale, (2) its use of an adaptive non-responder randomization design, (3) its use of actual behavior (i.e., clicks) to assess the primary outcome (i.e., MIBI adoption/preparation) and secondary outcome (i.e., MIBI exploration), and (4) its assessment of outcomes from both organization and individual perspectives. Several limitations should be noted. First, the researchers are the source (i.e., purveyor) of both the distributing educational materials and exploration facilitation strategies, as opposed to AETCs (i.e., intermediaries) being the source; it will not be known whether AETCs are a more effective source for these strategies. Second, the follow-up period will be limited to four-months post-dissemination, so it will be unknown whether implementation will be sustained beyond that period or whether some HSOs can achieve implementation if given a longer time. Third, MIBI adoption/preparation is operationalized as downloading of the MIBI manual and/or initiating the Tour of MI online asynchronous training, both of which may still be considered part of the exploration phase. Fourth, it is possible that a high rate of response during the one-week run-in period may impede our ability to deploy the exploration facilitation strategy. However, we believe the risk of this limitation is low.

### Trial status

The MOTIVATE CHANGE dissemination experiment received IRB approval on 10/15/2024, sent its initial distributing educational materials email on 10/29/2024, and ended on 5/14/25.

## Conclusion

Dissemination and implementation research is focused on improving the integration of EBIs within practice settings, and in turn improving public health. Currently, however, there is an imbalance, with much less dissemination research compared to implementation research, especially dissemination research employing experimental designs [25]. Beyond addressing this imbalance, the MOTIVATE CHANGE dissemination experiment will help advance generalizable knowledge about the extent to which an initial distributing educational materials strategy achieves MIBI adoption/preparation, and the extent to which a brief exploration facilitation strategy is an effective adjunct dissemination strategy to increase MIBI adoption/preparation. Ideally, findings from the MOTIVATE CHANGE dissemination experiment will inform what dissemination strategies the AETC network and other purveyors/intermediaries use for dissemination and also increase research interest in testing adaptive dissemination strategies.

## Supplementary Information

Below is the link to the electronic supplementary material.

Supplementary Material 1

Supplementary Material 2

Supplementary Material 3

## Data Availability

No datasets were generated or analysed during the current study.

## Abbreviations

AETC: AIDS Education and Training Center
EBI: Evidence-Based Interventions
EPIS: Exploration, Preparation, Implementation, Sustainment
HRSA: Health Resources and Services Administration
HSO: HIV Service Organization
ISF: Implementation and Sustainment Facilitation
IRB: Institutional Review Board
MI: Motivational Interviewing
MIBI: Motivational Interviewing Brief Intervention
MOTIVATE CHANGE: Motivational Outreach and Training Interventions for Vulnerable Affected Targeted Empowerment, and Counseling for HIV-positive clients with Addiction Needs for Guidance and Education
SUD: Substance Use Disorders
STAR: Strategies Timeline, Activities, and Rationale
