# Supplementary material for: Dissemination of an evidence-based motivational interviewing brief intervention for substance use disorders to HIV service organizations across the United States: protocol for a national-level cluster-randomized adaptive parallel-groups superiority experiment
Source: Addict Sci Clin Pract. 2025 Oct 23;20:85. doi: 10.1186/s13722-025-00612-8 (PMC12548271; doi:10.1186/s13722-025-00612-8)
Supplement: Supplementary file 1 — Supplementary Material 1 [file 13722_2025_612_MOESM1_ESM.pdf]

Supplemental File 1. Distribute Educational Material (DEM) initial email

MOTIVATE CHANGE

THE OHIO STATE UNIVERSITY
COLLEGE OF MEDICINE

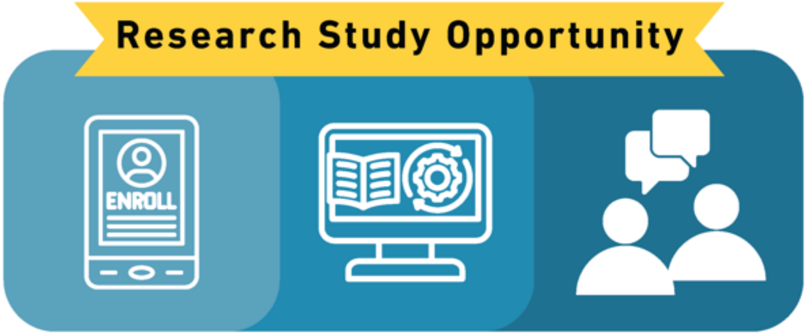

Learn Motivational Interviewing!

- Many people living with HIV also have a substance use disorder that interferes with their care.
- A one-time 15-30 minute Motivational Interviewing-Based Brief Intervention (MIBI) may reduce substance use and improve care.
- To help HIV Service Organizations (HSO) and their staff improve their capacity to address risky substance use among people with HIV, the MOTIVATE CHANGE Study is offering several training resources to help staff learn the MIBI.
- The purpose of this study is to offer HSOs the opportunity to learn **motivational interviewing** (MI) techniques for risky substance use among people with HIV and to assess adoption of MI at HSOs after four months.
- There is no cost to participate in this study.

Learn More & Enroll!

Funding for this project is provided by:
The National Institute On Drug Abuse
Principal Investigator: Bryan Garner,PhD
IRB Number: 2024B200

700 Ackerman Road, Suite 5000
Columbus, OH 43202
Unsubscribe | motivatechange@osumc.edu
