# Supplementary material for: Dissemination of an evidence-based motivational interviewing brief intervention for substance use disorders to HIV service organizations across the United States: protocol for a national-level cluster-randomized adaptive parallel-groups superiority experiment
Source: Addict Sci Clin Pract. 2025 Oct 23;20:85. doi: 10.1186/s13722-025-00612-8 (PMC12548271; doi:10.1186/s13722-025-00612-8)
Supplement: Supplementary file 3 — Supplementary Material 3 [file 13722_2025_612_MOESM3_ESM.pdf]

World Health Organization Trial Registration Data Set
(https://www.who.int/clinical-trials-registry-platform/network/who-data-set)

1. **Primary Registry and Trial Identifying Number:**
   Open Science Framework (OSF) and https://osf.io/grs68/

2. **Date of Registration in Primary Registry:**
   1/30/2025

3. **Secondary Identifying Numbers:**
   R01DA052294

4. **Source(s) of Monetary or Material Support:**
   The National Institute on Drug Abuse (R01DA052294)

5. **Primary Sponsor:**
   The Ohio State University

6. **Secondary Sponsor(s):**
   None

7. **Contact for Public Queries:**
   Bryan R. Garner, PhD (Principal Investigator)
   Professor and Director of Dissemination & Implementation Science
   The Ohio State University
   Bryan.Garner@osumc.edu

8. **Contact for Scientific Queries:**
   Bryan R. Garner, PhD (Principal Investigator)
   Professor and Director of Dissemination & Implementation Science
   The Ohio State University
   Bryan.Garner@osumc.edu

9. **Public Title:**
   The MOTIVATE CHANGE Dissemination Experiment

10. **Scientific Title:**
    Identifying and Disseminating Substance, Treatment, and Strategy (STS) Recommendations to AIDS Service Organizations

11. **Countries of Recruitment:**
    The United States of America

12. **Health Condition(s) or Problem(s) Studied:**
    Substance use disorders among people with HIV

13. **Interventions and/or Strategies:**
    Distribute Educational Materials (DEM) is the control strategy and Exploration Facilitation (EF) is the experimental strategy.

14. **Key Inclusion and Exclusion Criteria:**
    Eligibility criteria for the MOTIVATE CHANGE dissemination experiment are: (1) being a staff member of an agency in the HSO Directory located in the contiguous US states (due to limitations of gift card distribution, residents of Alaska and Hawaii were not eligible) or District of Columbia; (2) at least 18 years of age; (3) fluency in English, and (4) willingness and ability to provide consent to participate in the study.

15. **Study Type:**
Cluster-randomized adaptive parallel-groups superiority experiment

16. **Date of First Enrollment:**
10/29/2024

17. **Sample Size:**
750 HIV Service Organizations (HSOs) and 3,100 HSO staff.

18. **Recruitment Status:**
Complete

19. **Primary Outcomes:**
The primary outcome is adoption, defined as occurring when "the individual or organization engages in a number of activities that will lead to the research evidence being integrated into clinical practice and/or policy decisions." Per this definition and our cluster-randomized design, we will examine both staff-level MIBI adoption (i.e., HSO staff who download the MIBI manual and/or enroll in the online asynchronous motivational interviewing training) and organization-level MIBI adoption (i.e., HSOs with staff-level MIBI adoption by 1+ staff).

20. **Key Secondary Outcomes:**
Secondary outcomes are: staff-level MIBI exploration (i.e., HSO staff who click the "Learn More & Enroll!" button) and organization-level MIBI exploration (i.e., HSOs with staff-level MIBI exploration by 1+ staff).

21. **Ethics Review:**
The MOTIVATE CHANGE dissemination experiment was reviewed and approved by The Ohio State University IRB (Protocol 2024B0200), under Federalwide Assurance No. FWA00006378 from the U.S. Department of Health and Human Services' Office for Human Research Protections.

22. **Completion date:**
Planned for 4/1/2025

23. **Summary of Results:**
Pending

24. **IPD sharing statement:**
During the active data collection and analysis phase, access to data is restricted to the Principal Investigator, research coordinators, lead statistician, and statistical programmers. Following completion of the study and publication of primary findings, a public access dataset of de-identified data will be constructed. This public access dataset will be archived in the National Addiction & HIV Data Archive Program
